# Unusual intravesical foreign body in young female migrated from vagina due to autoerotism

**DOI:** 10.1590/S1677-5538.IBJU.2016.0164

**Published:** 2017

**Authors:** Ankur Bansal, Manoj Kumar, Gautam Kanodia, Ruchir Aeron, Sunny Goel

**Affiliations:** 1 King George Medical University, Lucknow, Uttar Pradesh, India

**Keywords:** Urinary Bladder, Vagina, Urogenital System

## INTRODUCTION

Foreign bodies are rarely found in genitourinary system and pose a challenge to the practitioner. The usual causes for insertion of foreign bodies in genitourinary system include sexual curiosity, autoerotic stimulation, or during invasive procedures (1). These patients may remain asymptomatic or have minimal discomfort but usually patient presents with urinary tract infection, severe pain and hematuria (2). Foreign bodies should be removed completely and procedures used should be simple and minimally traumatic to the genitourinary tract (1). Herein, we present a case and management of self-inserted foreign body in the vagina of a young girl for erotic stimulation.

## CASE PRESENTATION

**Figure 1:**
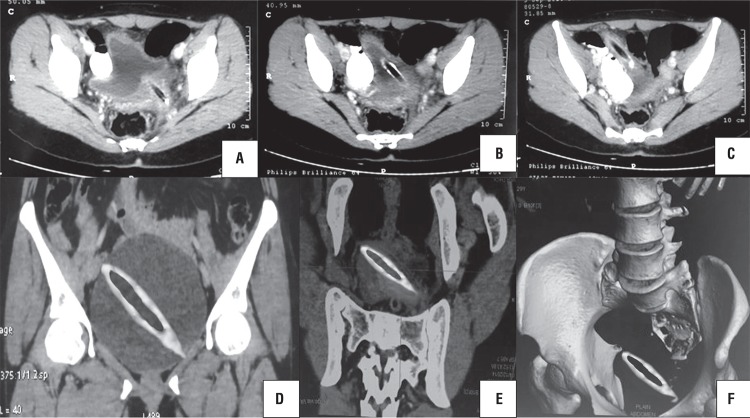
Computed tomography scan [axial section (A-C) and coronal section (D-F)] showing hypodense linear foreign body (10.2 x 1.2cm) piercing the anterior vaginal wall and left posterior bladder wall with majority of its part lying inside the bladder. The tip of foreign body pierced right to anterior bladder wall and reached the abdominal wall.

**Figure 2:**
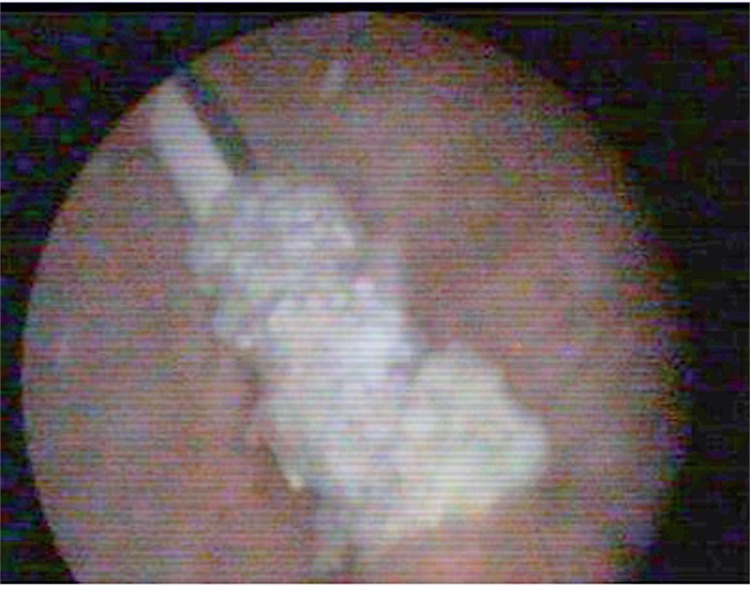
Cystoscopy showed encrusted plastic pen inside the bladder extending from right anterior bladder wall up to left posterior bladder wall.

**Figure 3:**
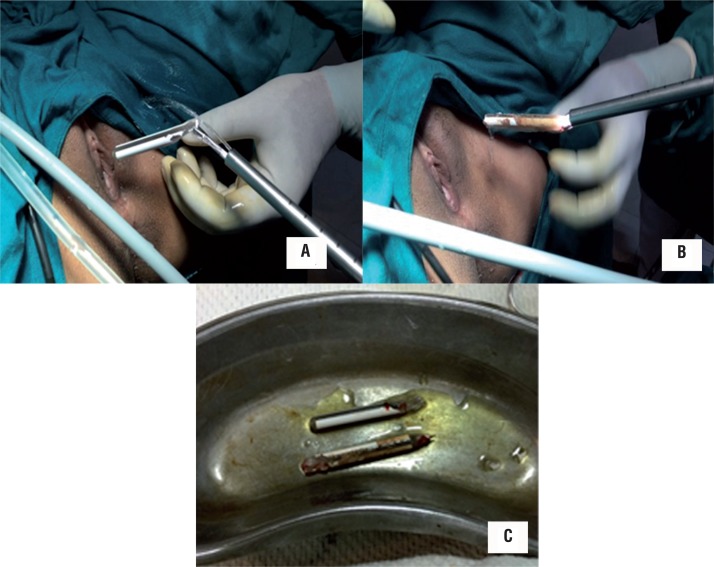
(A-C) - Foreign body (encrusted pen) was broken into two parts by transurethral cystolithopaxy using stone punch and was removed under cystoscopic guidance.

**Figure 4:**
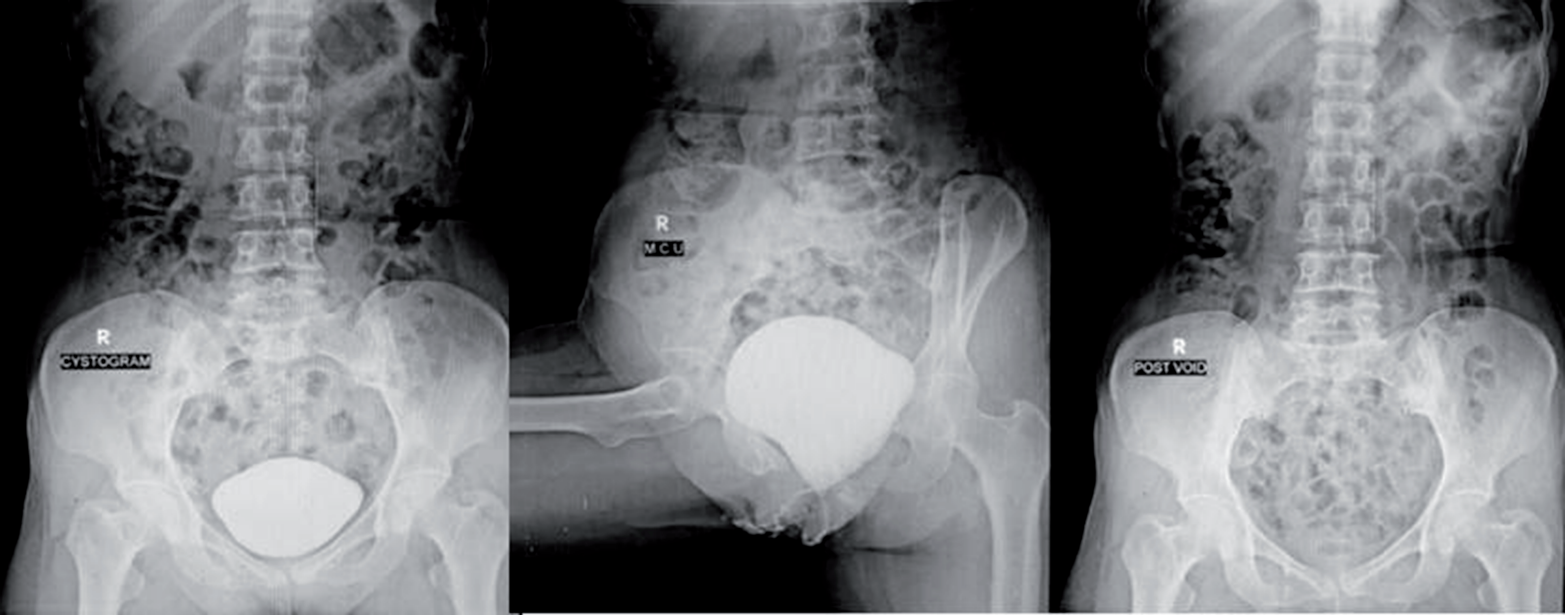
Voiding cystourethrogram revealed intact bladder and complete emptying of bladder in post void film with no dye in vagina.

An 18-year old unmarried illiterate girl presented with dysuria, increased frequency of micturition and occasional mild hematuria. The patient had history of insertion of plastic pen through vagina 6 months earlier for sexual gratification. There was no history of continuous leakage of urine per vagina. She informed history of normal menstruation. There was no associated psychiatric illness. Laboratory investigations such as electrolyte profile and blood count were normal but routine urine analysis showed pyuria and microscopic hematuria. General physical examination revealed no abnormality. On per vaginal and speculum examination, a pointed object was felt at anterior vaginal wall with no continuous leakage of urine from vagina. The digital rectal examination was normal. Plain X-ray pelvis was normal. Contrast enhanced computed tomography showed a 10.2 x 1.2cm hypodense linear object piercing the anterior vaginal wall and left posterior bladder wall with majority of its part lying inside the bladder. The tip of the foreign body pierced right to anterior bladder wall and reached the abdominal wall (Figure-1) with normal upper tracts, uterus and ovaries. Cystoscopy showed encrusted plastic pen inside the bladder extending from right anterior bladder wall up to the left posterior bladder wall (Figure-2) with a small portion of pen (approximately 3mm) protruding through anterior vaginal wall visualized on vaginoscopy. Patient refused psychiatric evaluation. Foreign body was broken into two parts by transurethral cystolithopaxy using stone punch under regional anesthesia and was removed under cystoscopic guidance (Figure-3). Following its removal, repeat cystoscopy and vaginoscopy revealed a 3 x 3mm supratrigonal vesicovaginal fistula with inflamed vaginal mucosa. Foley catheter (16Fr) was inserted per urethra and the patient was discharged on postoperative day 3 with an advice to follow-up after 3 weeks. She did not complain of continuous leakage of urine per vagina in the post-operative period. Foley catheter was removed at 3 weeks and voiding cystourethrogram was performed which revealed intact bladder and complete emptying of bladder in post void film with no dye in vagina (Figure-4). The patient was fully continent with no urine leakage per vagina. Patient was doing well at 6 months follow-up.

## DISCUSSION

Various intravesical foreign bodies reported include surgical gauze, pieces of Foley balloon catheter, intrauterine device, metal wire, carrot, lead pencil, ball pen, needle, household batteries, screw, pessaries and broken parts of endoscopic instruments etc. (3, 4). Multiple routes of entry of intravesical foreign bodies include self-insertion, iatrogenic, migration from adjacent organs, via urethra or traumatic route. Psychological circumstances which leads to self-insertion of such foreign bodies includes mental illness, sexual curiosity and borderline personality disorder (5). In our case, the reason of self-insertion was erotic stimulation and the route of insertion was traumatic migration from adjacent organ (vagina). Although the route of insertion mentioned and pointed out by the patient was vagina and not urethra, but as the patient was illiterate, it may not be accurate. Foreign bodies from adjacent viscera such as gastrointestinal tract and female genitourinary tract migrating traumatically into urinary bladder are extremely rare. In a study done by Rafique et al. (3), 5 such cases of foreign bodies migrating into bladder from genitourinary tract (intrauterine copper device in 4 females) and gastrointestinal tract (3-inch copper wire being swallowed by young boy) were reported. Our case was very interesting and is probably the first case report in which the pen inserted in vagina for sexual gratification almost completely migrated into bladder.

Usually patient remains asymptomatic or may present with symptoms related to irritation of the lower urinary tract such as frequency, dysuria, microscopic or gross haematuria, lower abdominal pain, urethral discharge, strangury and acute urinary retention (2, 3). During sexual history or urogenital examination if the patient becomes anxious, high suspicion for self-insertion of foreign bodies should always be kept in mind (3).

Radiologic evaluation helps in determining the exact size, location and number of the foreign bodies (1). Confirmation can easily be done in cases of radiopaque foreign bodies with plain kidney urinary bladder (KUB) radiograph and for radiolucent foreign bodies with ultrasound and computed tomography (CT) (6). However, urethrocystoscopy remains the most accurate method for diagnosis of intravesical foreign bodies.

Nowadays, endoscopic procedures are preferred treatment modalities as they minimize the lower urinary tract injuries. However, open procedures like suprapubic cystostomy are still recommended in few cases to reduce the risk of urethral and bladder injury (1). As female bladder can be easily accessed via urethra, foreign bodies can safely be removed endoscopically (4). Due to high incidence of psychiatric disease, dementia and mental retardation in these patients, routine psychiatric evaluation is recommended (7). Although it is not universally accepted, this will prevent further incidence of insertion of foreign bodies in genitourinary tract.

Urogenital fistula can be a complication of foreign body insertion in genitourinary tract. Management of urogenital fistulas depends on size and location of the defect. Spontaneous healing can occur with bladder drainage alone if the fistula size is small. Davits et al. (8) reported a series of four patients in whom fistula developed after vaginal and abdominal hysterectomy, and treated successfully with prolonged bladder drainage (19-54 days). Spontaneous closure of the fistula is unlikely if healing does not occur within 4 weeks (9).

Conclusion: In young patients presenting with chronic lower urinary tract symptoms, foreign bodies should always be kept in mind as a differential diagnosis. Detailed history and clinical examination can detect the presence of a foreign body, however imaging modalities like X-ray pelvis, CT whole abdomen and endoscopy (cystoscopy/vaginoscopy) may be required. With advancement in endoscopic techniques, majority of cases can be treated successfully with minimally invasive techniques. Small vesicovaginal fistulas are likely to heal spontaneously with prolonged catheterization.
